# Editorial: Emerging human viruses with pandemic potential: Diagnostics, pathogenesis, and therapeutics

**DOI:** 10.3389/fcimb.2023.1182522

**Published:** 2023-03-23

**Authors:** Viviane Fongaro Botosso, Edison Luiz Durigon, Edmarcia Elisa de Souza

**Affiliations:** ^1^ Virology Laboratory, Butantan Institute, São Paulo, Brazil; ^2^ Scientific Platform Pasteur-University of São Paulo, São Paulo, Brazil; ^3^ Department of Microbiology, Institute of Biomedical Sciences, University of São Paulo, São Paulo, Brazil; ^4^ Unit for Drug Discovery, Department of Parasitology, Institute of Biomedical Sciences, University of São Paulo, São Paulo, Brazil

**Keywords:** emerging viruses, diagnostics, pathogenesis, therapeutics, epidemiology

The emergence of viral pathogens and their subsequent spreading have caused an extremely significant impact on human health and the global economy. Most notably, the COVID-19 pandemic posed an enormous threat to public health worldwide, both because of its pathological characteristics in addition to high transmissibility and rapid evolution of SARS-CoV-2. Considering this, studies focused on biology, pathogenesis, epidemiology, diagnosis and prevention of viral pathogens are fundamental to control future emerging infectious diseases. In this Research Topic, we received 19 manuscripts, of which 9 were accepted for publication after rigorous peer review processes. We thank all the authors and reviewers for their valuable contributions, and we expect that this article collection will be helpful for the scientific community seeking knowledge about emerging viruses.

Andrade et al., present a comprehensive review highlighting the homeostatic alterations caused by SARS-CoV-2 infections. In general, hypercoagulation, endothelial dysfunction and dysregulation of the renin-angiotensin system are important determinants for pulmonary thrombus formation and impairment of respiratory functions observed in patients with severe COVID-19. Chen et al., report that ferroptosis, a cell death mechanism characterized by iron overload and lipid peroxidation, may participate in SARS-CoV-2 infection associated liver injury, a common feature in COVID-19. Potential links between ferroptosis and COVID-19 are associated with higher frequencies of hepatic steatosis, Kupffer cell activation, vascular thrombosis, and inflammatory infiltration. However, it is still unclear how ferroptosis drives these pathological processes contributing to liver injury caused by SARS-CoV-2. Additionally, Yang et al., emphasis the roles of non-coding RNAs (ncRNAs) in neurological complications induced by Enterovirus 71 (EV71). In this respect, host ncRNAs target EV71 genome to promote invasion and modulate its replication; this event may damage key signaling pathways of central nervous system, resulting in acute immune and inflammatory responses. Essentially, these reports provide a basis for the mechanisms that contribute to pathological features of COVID-19 or EV71-associated diseases.

Understanding risk factors and serological markers that can influence the progression of the disease are crucial for treatment and prevention measures. Under these premises, Queiroz et al., evaluate the main risk factors correlated with the severity and progression of COVID-19. In this study, the assessment of clinical manifestations of patients infected with SARS-CoV-2 demonstrated that elevated cytokine levels among individuals with severe acute COVID-19 is associated to sex, advanced age, and presence of comorbidities such as diabetes mellitus, hypertension, chronic kidney disease, obesity, and immunosuppression. In addition, it was possible to identify cytokine markers that are characteristic for disease progression to long COVID-19. Similarly, Torres et al., provide a detailed study of risk factors associated to SARS-CoV-2 seroprevalence at first wave in comparison with the second wave of COVID-19 in the city of Beleím, state of Paraí, northern Brazil. According to this study, behavioral profiles including the frequency of travel, low frequency of protective mask use, hygiene habits, lack of social isolation, and contact with infected people, in addition to socioeconomic discrepancies as low education level, are considered risk factors for SARS-CoV-2 infection. In addition, Chen et al., present an interesting research manuscript regarding the predictability of mortality of COVID-19 patients. The prognostic model demonstrated that age and high levels of UREA and lactate dehydrogenase (LDH) were associated with mortality of 80-days COVID-19 patients, suggesting a robust tool for predicting mortality and assist clinicians in the early screening of patients with COVID-19 poor prognoses.

Hu et al., and Yang et al., highlight the need to the development of tools that can be applicable to early diagnosis of COVID-19 and HIV infection, respectively. Hu et al., report a RNA fluorescence *in situ* hybridization (FISH) method that detects SARS-CoV-2 spike (S) and envelope (E) proteins and their mRNAs, with enhanced signal of fluorescence generated within a hybridization reaction inside HEK 293T cells. Yang et al., evaluate the performance of Elecsys^®^ HIV Duo assay for diagnostic of HIV/AIDS from clinical patient samples in southwest China. This study demonstrated the detection of the earliest immune markers HIV-1 p24 antigen and HIV-1/2 antibody simultaneously, which greatly enhanced the performance of test results. These approaches might improve the detection sensitivity and specificity and support the early diagnosis of SARS-CoV-2 and HIV, respectively.

While several emerging viruses have caused outbreaks, detailed knowledge of their behavior and habitats are essential to predict potential outbreaks and spillovers of zoonotic diseases (Harvey and Holmes, 2022). Regarding to that, Loh et al., present a study of viral diversity in bat host species in deforested versus forested areas of the Atlantic Forest of Brazil. Overall, the study demonstrated the prevalence of high viral richness in active deforestation sites, which may result in increased risk to human exposure with zoonotic infections and disease reservoirs.

## Author contributions

VB, ED and ES wrote and edited the manuscript. All authors contributed to the article and approved the Editorial.
